# Concurrent isolation of hepatic stem cells and hepatocytes from the human liver

**DOI:** 10.1007/s11626-020-00433-w

**Published:** 2020-03-27

**Authors:** Serene M. L. Lee, Cristina Bertinetti-Lapatki, Tobias S. Schiergens, Karl-Walter Jauch, Adrian B. Roth, Wolfgang E. Thasler

**Affiliations:** 1 Department of General, Visceral and Transplant Surgery, Ludwig-Maximilians-University Munich, 5H 02 Room 428, Marchioninistr. 15, 81377 Munich, Germany; 2 F. Hoffmann-La Roche Ltd, Pharmaceutical Sciences, Roche Innovation Centre, Grenzacherstr 124, 4070 Basel, Switzerland; 3 Medical Directorate, Ludwig-Maximilians-University Munich, Marchioninistr. 15, 81377 Munich, Germany; 4 Department of General Visceral and Minimally Invasive Surgery, Red Cross Hospital Munich, Nymphenburger Str. 163, 80634 Munich, Germany

**Keywords:** Hepatic stem cells, Hepatic progenitor cells, Hepatocytes, Liver cell isolation, Liver *in vitro* model

## Abstract

Hepatocytes differentiated from induced pluripotent stem cells or stem cells have the potential to be representative *in vitro* models of the human liver for research as well as early safety assessment programs. However, up until now, there has been no definitive proof that differentiated hepatocytes recapitulate the phenotype and functional characteristics of primary hepatocytes from the same individual. Thus, a method for the concurrent isolation of hepatocytes and hepatic stem cells is presented here to provide the cells necessary for the evaluation of the required benchmarking. The method presented here generated high-quality hepatocytes with a purity of 94 ± 1% and a high percentage viability of 79 ± 2%. Furthermore, the hepatic stem cells isolated were found to be actively proliferating and have a purity of 98 ± 1%. Thus, these isolated cells can be used as a powerful tool for the validation of differentiated hepatocyte *in vitro* models.

## Introduction

The cost of bringing a new drug to market has skyrocketed mainly due to the outlay on phase 2 and 3 trials and late-stage failures (Orloff et al. 2009). Reducing late-stage drug attrition costs can best be done by early safety assessment programs. Currently, human primary hepatocytes are used as the “gold standard” *in vitro* model in such programs. While this model has many advantages, its usage also brings substantial challenges in a pharmaceutical environment. These challenges include limited quantity available, lack of availability when required, variable quality of hepatocytes dependent on the tissue quality or isolation procedures, uncharacterised enzymatic profiles when cells are received and long waiting times for a specific donor profile. Thus, it is very important to establish an equivalent *in vitro* human model without these disadvantages.

The use of hepatocytes differentiated from induced pluripotent stem cells (iPSC) could be an ideal solution as such hepatocytes could potentially provide unlimited *in vitro* models with pre-characterised and desired phenotypic backgrounds. Moreover, it offers the opportunity to include different disease phenotypes in drug toxicity testing. However, despite many attempts over the years to generate hepatocytes from iPSCs (iPSC-Heps), no iPSC-Heps representative of donor phenotype has been produced to our knowledge.

One of the reasons for this difficulty is obtaining a mature and stably differentiated phenotype. Generating iPSCs from Hepatic Stem Cells (HSCs) could help with this problem. Hepatic stem cells in this text refer to self-renewing cells that have the potential to give rise to both hepatocytes and cholangiocytes (Schmelzer et al. 2007). The actual nomenclature to be used, whether the term HSC or hepatic progenitor cell is still unclear and controversial in the literature. The reasons that we choose to isolate HSCs in particular are manifold. Firstly, iPSCs generated from bipotential HSCs could retain a transient transcriptional memory that facilitates differentiation into hepatocytes especially at lower passage numbers (Lee et al. 2012). Secondly, unlike hepatocytes, HSCs can proliferate in culture and this could promote the formation of induced pluripotent stem cells (Panopoulos et al. 2012). Thirdly, if reprogramming to iPSCs is not desired, hepatocytes can be directly differentiated from HSCs, which have been shown to be able to persist in culture for up to 100 passages over 2 yr (Wang et al. 2010). The ability to keep HSCs in long-term culture will also allow investigators to examine differences due to reprogramming and the influence of long-term culture with or without reprogramming on subsequent differentiation to hepatocytes. Previously published works show that Epithelial Cell Adhesion Molecule (EpCAM), a transmembrane glycoprotein, is an extracellular marker expressed by HSCs (Schmelzer et al. 2007; Okabe et al. 2009) and this marker will be targeted for the isolation of HSCs in this work.

The other major reason that has made it impossible to say if differentiated hepatocytes have been successfully produced was the lack of primary hepatocytes from the same donor for benchmarking. Until today, almost all previous studies have compared functional properties of differentiated hepatocytes, such as cytochrome P450 enzyme activities, against other less relevant cell types such as undifferentiated iPSCs, early stages of differentiating stem cells or hepatic cell lines (Ulvestad et al. 2013). However, this strategy of comparing against cell types with little or no mature hepatocyte characteristics does not allow a conclusion that the donor phenotype as seen in the primary hepatocytes has been recreated.

Therefore, this study aims to provide a powerful tool to researchers to address these issues by providing a method to isolate HSCs and hepatocytes from the same donor.

## Materials and Methods

### Isolation of hepatocytes and hepatic stem cells

Pieces of macroscopically normal liver with 1 sectioned surface from donors undergoing elective surgeries for secondary metastasis to the liver were included in this study. The liver samples were provided double-coded by the Biobank under the administration of the Human Tissue and Cell Research (HTCR) Foundation at the Ludwig-Maximilians-University (LMU). The framework of the HTCR Foundation, which includes written informed consent from all donors, has been approved by the ethics commission of the Faculty of Medicine in the LMU (number 025-12) and the Bavarian State Medical Association (number 11142).

A modified (Lee et al. 2013) two-step collagenase perfusion technique (Lee et al. 2013; Berry and Friend 1969; Seglen 1973) was used to prepare a cell suspension containing human hepatocytes and HSCs. In brief, irrigation cannulas with olive tips were inserted snugly into the larger blood vessels on the cut face of the piece of the liver for perfusion at 37°C. The liver was perfused sequentially with perfusion buffer (154 mM sodium chloride, 20 mM HEPES, 5.6 mM potassium chloride, 5 mM glucose and 25 mM sodium hydrogen carbonate), perfusion buffer containing 1 mM EGTA, perfusion buffer containing 5 mM calcium chloride dihydrate and finally perfusion buffer containing 5 mM calcium chloride dihydrate and 250 to 400 U ml^−1^ collagenase (Nordmark Biochemicals, Uetersen, Germany). Enzymatic digestion with collagenase was carried out in a recirculating manner for 9 to 12 min or until the liver is sufficiently digested. The liver piece was then placed carefully in a crystallising dish for removal of the Glisson’s capsule before gently shaking the cells loose. The cell suspension was then filtered through a 210-μm nylon mesh followed by a 70-μm nylon mesh.

After filtration, the cell suspension was centrifuged at 72*g* for 5 min at 4°C. The first supernatant obtained, which contained the HSCs, was transferred to new centrifuge tubes and set on ice for the subsequent isolation of HSCs. The hepatocyte pellet was resuspended in wash buffer (120 mM sodium chloride, 10 mM HEPES, 0.9 mM calcium chloride dihydrate, 6.2 mM potassium chloride and 0.1% *w*/*v* albumin) for hepatocyte isolation.

To complete hepatocyte isolation, the hepatocytes were washed by centrifugation at 72*g* for 5 min at 4°C followed by aspirating off the wash buffer in the supernatant and topping up the centrifuge tube with fresh wash buffer. The above washing step was repeated twice. On the last centrifugation step, the hepatocytes were resuspended in the appropriate cell culture media for a haemocytometer-based trypan blue exclusion assay to determine hepatocyte yield and viability. At this stage, the purified hepatocytes can be used in suspension or adhered to collagen-coated plates for further experiments. For adherence to 6-well Biocoat™ collagen 1-coated plates (Corning, Corning, NY), 1.2 million hepatocytes in hepatocyte cell culture media were added per well. These hepatocytes were allowed to adhere for 4 h in an incubator at 37°C. Thereafter, a media change was carried out to remove any non-adhered cells. The hepatocyte culture medium used was DMEM media supplemented with 800 μg/l hydrocortisone, 125 U/l insulin, 100 U/ml penicillin, 100 μg/ml streptomycin, 2 mM l-glutamine, 7.4 μg/l glucagon and 10% FCS.

To complete HSC isolation from the supernatant generated by the first centrifugation, the remaining contaminating hepatocytes had to be removed. This was done by centrifuging at 100*g* for 5 min at 4°C and transferring the supernatant to new centrifuge tubes at least twice or until no hepatocyte pellet can be seen after centrifugation. The supernatant free of hepatocytes was then centrifuged at 300*g* for 5 min at 4°C to obtain a non-parenchymal cell pellet containing HSCs. The cell pellet was resuspended in a volume of degassed cell suspension buffer (Phosphate Buffered Saline (PBS) containing 2 mM Ethylenediaminetetraacetic Acid (EDTA) and 0.5% *w*/*v* Bovine Serum Albumin (BSA)) at least equal to the volume of the pellet before carrying out red blood cell lysis using a kit from Miltenyi Biotec (Teterow, Germany) according to the manufacturer’s instructions. After red blood cell lysis, the cells were resuspended in cell suspension buffer and centrifuged at 300*g* for 5 min at 4°C. After resuspension in a small volume of cell suspension buffer, the cell suspension was filtered through a 70-μm cell strainer (Miltenyi Biotec) and counted using a haemocytometer.

Next, magnetic-activated cell sorting (MACS) was carried out to positively select Epithelial Cell Adhesion Molecule (EpCAM)-expressing HSCs. There have been many markers used for HSC isolation in a variety of species. For this article, EpCAM was chosen as it has been shown to be consistently expressed in normal liver tissue (Schmelzer et al. 2007; Okabe et al. 2009). Briefly, cells were resuspended to a concentration of 50 million total cells per 0.3 ml of cell suspension buffer before adding 0.1 ml of FcR Blocking reagent (Miltenyi Biotec) followed by 0.1 ml of EpCAM microbeads (Miltenyi Biotec) for each 50 million portion of cells. After swirling the cell suspension, the mixture was incubated at 4 to 8°C for 30 min. After the incubation period, cells were washed in cell suspension buffer and resuspended with up to 200 million cells ml^−1^ cell suspension buffer before carrying out magnetic selection. Magnetic selection was carried out using the MiniMACS™ Separator with MS Columns (Miltenyi Biotec) according to the manufacturer’s instructions.

After magnetic selection, the purified HSC suspension was centrifuged at 300*g* for 5 min at 4°C. The pellet was resuspended in oval cell culture medium (William’s Medium E containing 10% FCS, 10 mM nicotinamide, 2 mM l-glutamine, 0.2 mM ascorbic acid, 20 mM HEPES, 1 mM sodium pyruvate, 17.6 mM sodium bicarbonate, 14 mM α-D-glucose, 100 nM dexamethasone, 1X Insulin-Transferrin-Selenium, 0.5 μg ml^−1^ gentamicin, 10 ng ml^−1^ recombinant human epidermal growth factor, 10 ng ml^−1^ recombinant human hepatocyte growth factor and 10 ng ml^−1^ interleukin 6) and adhered on Corning™ Biocoat™ collagen 1-coated plates for further experiments (Okabe et al. 2009).

### Immunofluorescent staining

Ten-micrometer cryosections on microscope slides (Marienfeld-Superior, Lauda-Königshofen, Germany) or cells adhered on 8-well culture slides coated with collagen were stained using immunofluorescence. In brief, the specimens were fixed for 10 min in 10% neutral-buffered formalin and subsequently permeabilised using 0.5% triton in PBS. After blocking with PBS containing 10% goat serum and 0.1% triton for a minimum of 1 h at room temperature, specimens were incubated with primary antibodies at 4°C overnight. Primary antibodies used were against Cytokeratin 19 (CK19) (PA5-29582, Thermo Fisher Scientific, Waltham, MA), albumin (PA5-27707, Thermo Fisher Scientific) and Ki-67 (ab15580, Abcam, Cambridge, UK). The primary antibodies were used at a dilution of 1:100, 1:100 and 0.5 μg antibody ml^−1^ respectively. The next day, the specimens were incubated with the secondary antibodies conjugated with Alexa Fluor 488 (A-11034, Thermo Fisher Scientific) and 594 (A-11020, Thermo Fisher Scientific) for 1.5 h. The 2 secondary antibodies were used at a dilution of 1:500. Finally, cover slips were mounted using Prolong Gold Antifade Mountant (Thermo Fisher Scientific).

### CYP1A2 activity measurement

CYP1A2 activity was determined using the P450-Glo CYP1A2 luminescent assay kit (Promega Corporation, San Luis Obispo, CA) according to the manufacturer’s instructions.

### Fluorescence-activated cell sorting

To prepare cells for analysis, cells were fixed with 10% neutral-buffered formalin for 10 min at 37°C followed by chilling on ice for 1 min. Next, cells were permeabilised by the slow addition of ice-cold 100% methanol to a final concentration of 90% methanol before incubation on ice for 30 min. Cells were then blocked in PBS containing 0.5% BSA for 10 min at room temperature. Incubation of the cells with the primary anti-albumin antibody (PA5-27707, Thermo Fisher Scientific) at 5 μg antibody ml^−1^ was done for 1 h followed by 30-min incubation with a secondary antibody conjugated to Alexa Fluor 488 (A-11034, Thermo Fisher Scientific) at 5 μg antibody ml^−1^. Washing with PBS containing 0.5% BSA was carried out in between the key steps mentioned above.

Fluorescence-activated cell sorting (FACS) was carried out using a FACSCalibur (BD Biosciences, Heidelberg, Germany) and data collected was analysed using FlowJo version 10 (Flowjo LLC, Ashland, OR).

### **Statistical analysis**

Values are presented as means ± standard error of the mean. Two-tailed paired *t* tests done using Spss Statistics (IBM, Armonk, NY) were used to identify significant differences (*P* < 0.05) between means.

## Results

### CK19^+^ cells express EpCAM in human normal and cirrhotic liver

**Figure 1.**
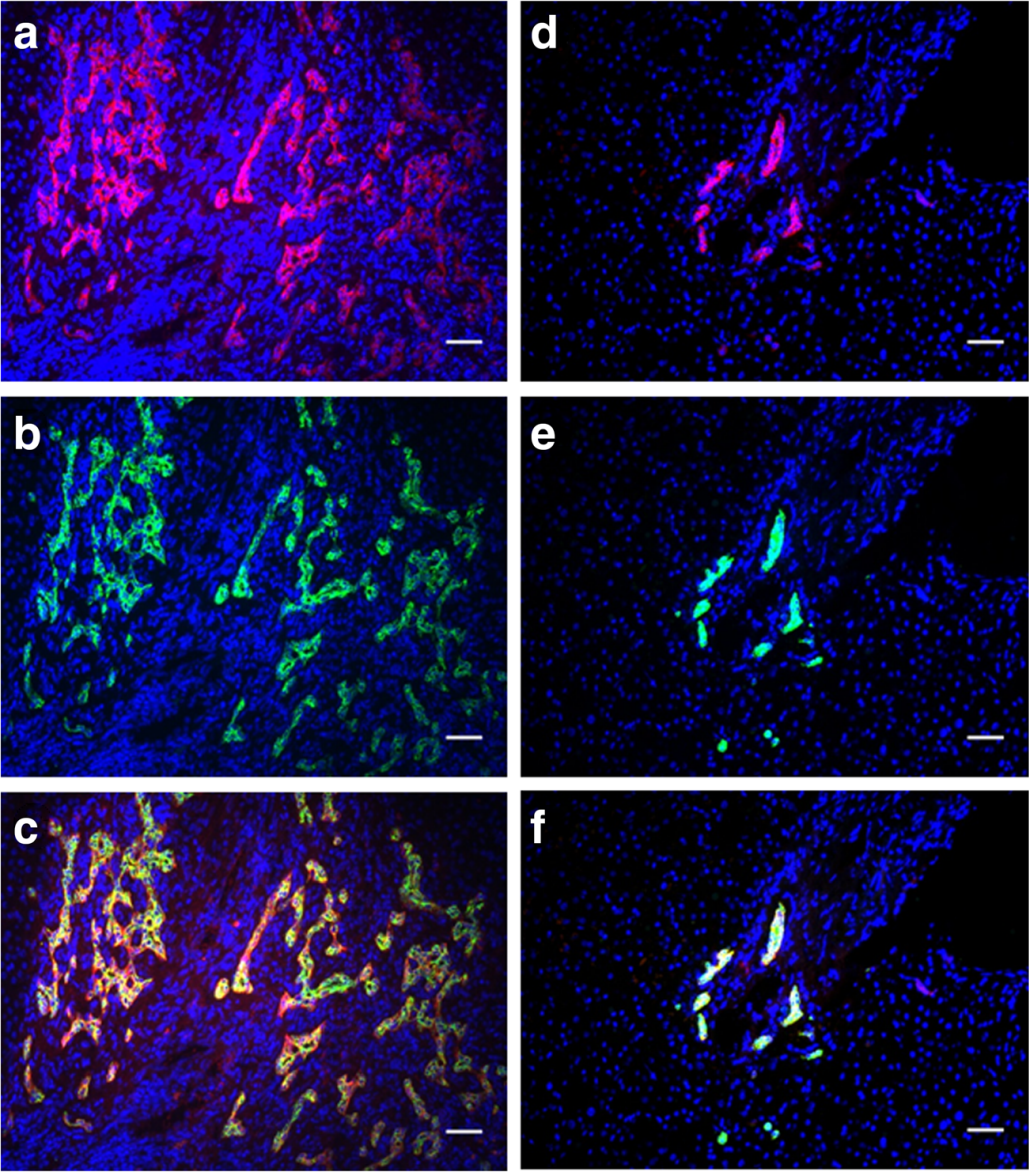
EpCAM and CK19 colocalise in human liver. Immunofluorescent staining of EpCAM and CK19 in cirrhotic (*a*–*c*) or in macroscopically normal liver (*d*–*f*). EpCAM localisation is visualised in red in panels (*a*) and (*d*), while CK19 localisation is visualised in green in panels (*b*) and (*e*). Co-localised expression of EpCAM and CK19 can be seen in panel (*c*) for cirrhotic liver and panel (*f*) for normal liver. Scale bars in the figure indicate a length 100 μm.

In order to validate the usage of EpCAM^+^ as a selection marker for human HSCs in our laboratory, expression was examined in human normal and cirrhotic liver cryosections (Fig. 1). Similar to the DDC-fed mice, an increase in ductular structures formed by EpCAM^**+**^ cells was seen in human cirrhotic liver (Fig. 1*a*). All these EpCAM^+^ cells also expressed Cytokeratin 19 (CK19 (Fig. 1*b*)), a marker of HSCs and cholangiocytes. This marker expression pattern was also seen in normal liver, albeit with markedly less ductular structures present when compared with the cirrhotic liver (Fig. 1*d*–*f*). Thus, similar to the mouse model, all human HSCs that express CK19 also expressed EpCAM (Fig. 1*c*, *f*), meaning that EpCAM can be used as the selection marker.

### Characterisation of isolated hepatocytes

**Figure 2.**
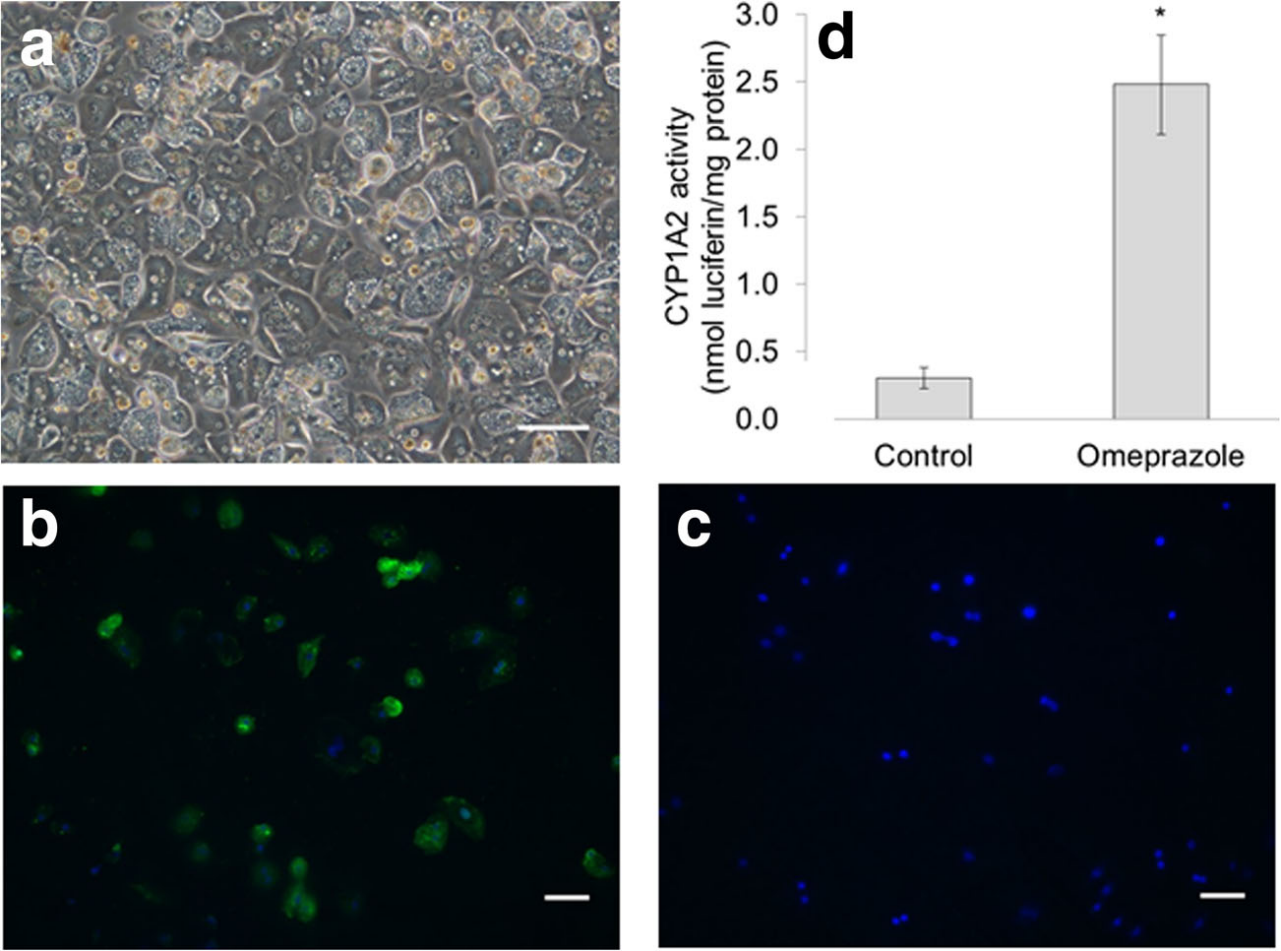
Hepatocyte characterisation.(*a*) Representative phase contrast image of hepatocytes adhered to collagen 1-coated plate 2 d after isolation. (*b*) Representative immunofluorescent image showing characteristic albumin (green) staining in hepatocytes with (*c*) corresponding secondary antibody control image. Nuclei were counterstained blue using DAPI. (*d*) CYP1A2 activity was induced in hepatocytes after treatment with 100 μM omeprazole for 48 h. Values from 3 biological replicates are presented in the *histogram*. *Significantly different from control, *P* < 0.05. *Scale bars* in the figure indicate a length 100 μm.

Characterisation of hepatocytes isolated in parallel with HSCs shows that the isolated hepatocytes had a high average viability of 79 ± 2% with a yield of 5 ± 1 million hepatocytes per gram liver (*N* = 11). Furthermore, phase contrast microscopy showed that the isolated hepatocytes exhibited key characteristics of hepatocytes; isolated cells were large polygonal cells with numerous binucleate cells (Fig. 2*a*). In addition, immunofluorescent staining showed that these isolated cells expressed hepatocyte marker albumin (Fig. 2*b*). Finally, these cells expressed a basal activity of CYP1A2 (0.31 ± 0.08 nmol luciferin mg^−1^ protein), which could be induced after treatment with 100 μM of omeprazole to 2.48 ± 0.37 nmol luciferin mg^−1^ protein for 2 d (Fig. 2*d*). Thus, hepatocytes isolated using this method are suitable for further experiments.

### Characterisation of isolated hepatic stem cells

This isolation method yielded 1.2 ± 0.2 million live non-parenchymal cells per gram liver (*N* = 12) for the subsequent isolation of EpCAM^+^ cells. After carrying out MACS, the yield of positively selected EpCAM^+^ cells was 15,076 ± 4307 cells per gram liver (*N* = 12). Since relatively large pieces of liver were used for cell isolation, the total yield of 0.86 ± 0.21 million EpCAM^+^ cells was sufficient for further experiments.

**Figure 3.**
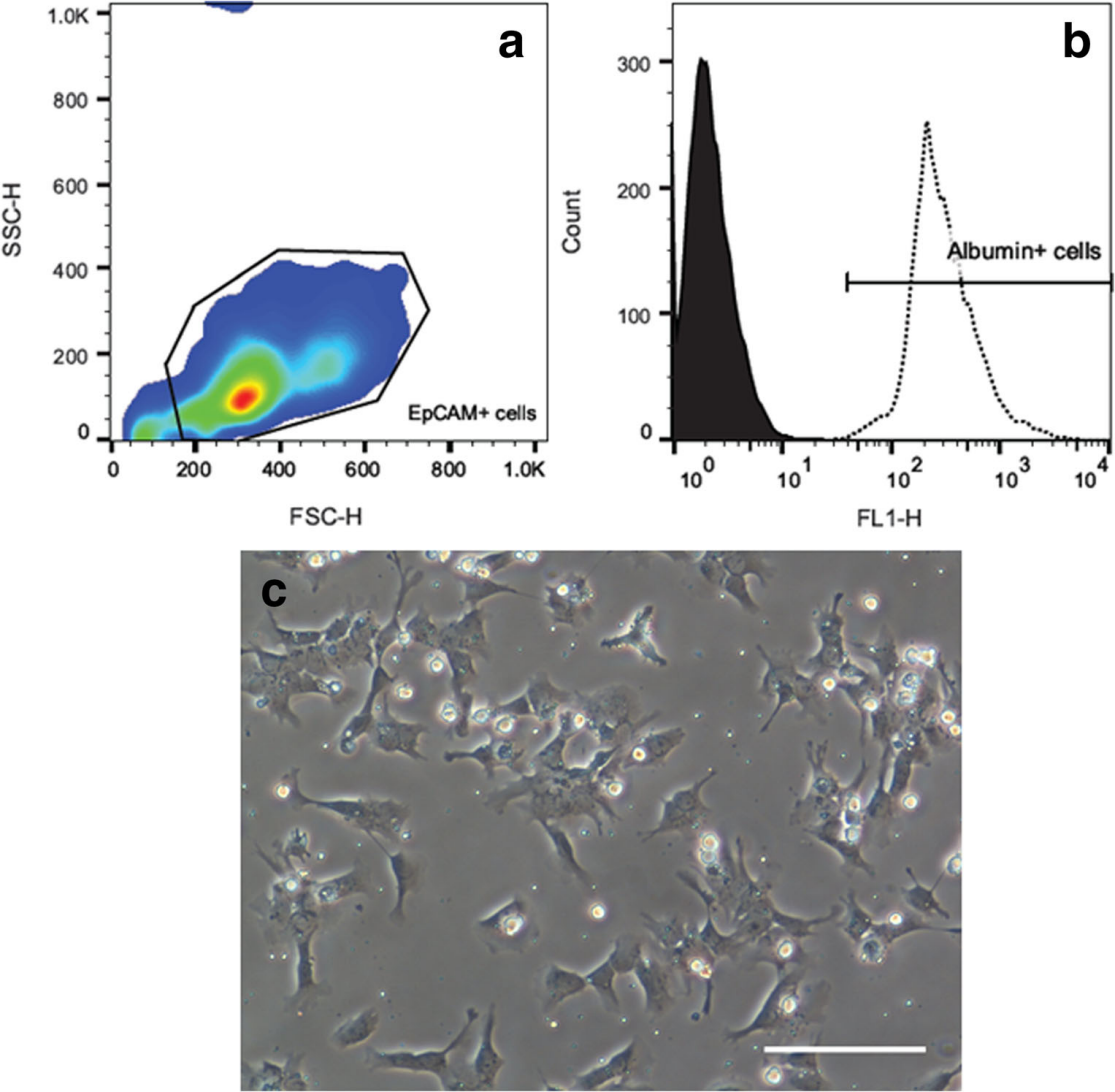
Characterisation of EpCAM^+^ cells immediately after isolation. (*a*) Representative pseudocolor plot showing the forward and side scatter of the isolated EpCAM^+^ cells. (*b*) Overlaid *histograms* showing control/unstained cells (*filled black*) and albumin^+^ cells (*dotted line*). Ninety-eight percent of the isolated EpCAM^+^ cells expressed albumin, a marker expressed by hepatic stem cells. (*c*) Representative phase contrast image of hepatic stem cells 1 d after isolation showing characteristic oval nuclei and scant cytoplasm. The *scale bar* in the figure indicates a length 100 μm.

**Figure 4.**
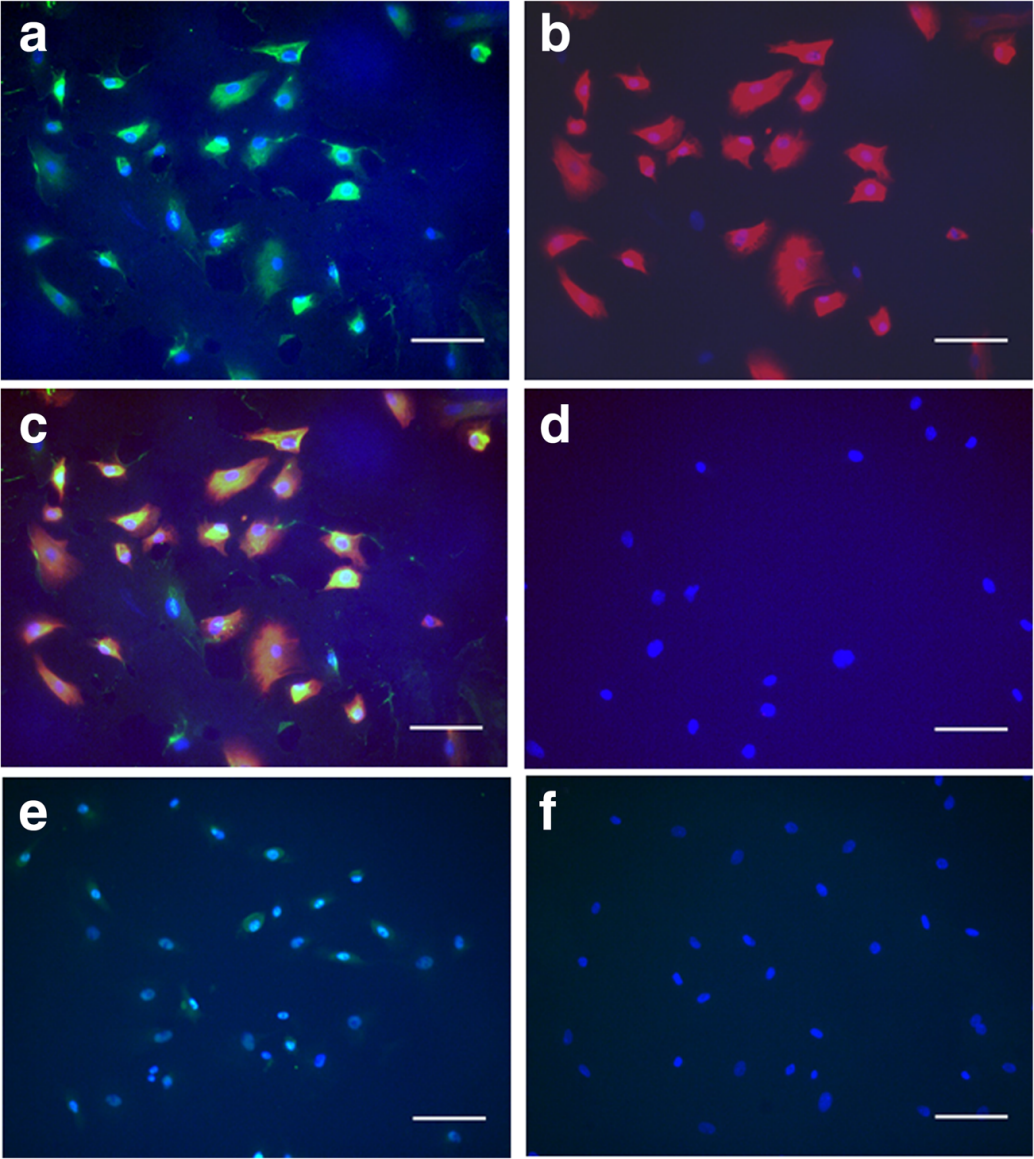
Hepatic stem cell characterisation. Immunofluorescent labelling of (*a*) albumin (green) and (*b*) CK19 (red) in hepatic stem cells 5 d after isolation with the co-localised expression of albumin and CK19 shown in panel (*c*). The corresponding secondary antibody control image is shown in panel (*d*). These cells express (*e*) proliferation marker Ki67 (green) in the nuclei making them appear cyan when compared with the corresponding (*f*) secondary antibody control with blue nuclei. Nuclei are counterstained blue with DAPI. Scale bars in the figure indicate a length 100 μm.

Immediately after isolation, EpCAM^+^ cells were characterised by FACS. Figure 3*a* shows the front and side scatter of the population of EpCAM^+^ cells. When examined for albumin expression, 98 ± 1% of the cells were found to express this marker (Fig. 3*b*). Since we have established that EpCAM^+^ cells also express CK19, the co-expression of CK19 and albumin indicates that the isolated cells are a pure population of HSCs. Phase contrast images taken 1 d after isolation support this assertion as it can be seen that the isolated cells have ovoid nuclei and scant cytoplasm characteristic of HSCs (Fig. 3*c*). Furthermore, cultured cells maintained stem cell phenotype on day 5 of culture as evidenced by the co-expression of albumin (Fig. 4*a*) and CK19 (Fig. 4*b*) in Fig. 4*c*. In addition, all of these cells expressed Ki67 (Fig. 4*e*), a marker of proliferation. Cell proliferation is of note as it could promote the formation of iPSCs (Panopoulos et al. 2012), which is a common goal for many researchers who work with HSCs.

## Discussion

Okabe *et al.* (Okabe et al. 2009) published a simple FACS-based method for the isolation of mouse HSCs through the selection of EpCAM^+^ cells in normal and injured liver. Their publication reported that the yield of HSCs from mouse livers injured by a 3,5-diethoxycarbonyl-1,4-dihydro-collidine (DDC)-containing diet was more than 2-fold compared with normal livers (Okabe et al. 2009). DDC is a porphyrinogenic agent that causes chronic oxidative liver damage, hepatocyte ballooning and the formation of Mallory-Denk bodies (De Matteis et al. 1973; Snider et al. 2011). Further evidence that EpCAM is a suitable isolation marker comes from Schmelzer *et al.* (Schmelzer et al. 2007), who have used EpCAM selection to isolate HSCs from neonatal, pediatric and adult livers with the finding that neonatal and pediatric livers yield 2- to 3-fold more EpCAM^+^ cells. Although a lower HSC yield from normal livers has been reported by the above authors (Schmelzer et al. 2007; Okabe et al. 2009), we believe that it is important to optimise and present a method to isolate HSCs and hepatocytes from normal human livers using EpCAM marker selection. One of the reasons for this importance is that although many authors have generated iPSCs from dermal fibroblasts and peripheral blood cells with subsequent differentiation to hepatocytes, these studies either did not compare the iPSC-Heps to primary human hepatocytes or were not able to compare the iPSC-Heps to primary hepatocytes from the same donor (Ghodsizadeh et al. 2010; Kajiwara et al. 2012; Krueger et al. 2013; Ulvestad et al. 2013). With the aim of isolating HSCs and primary hepatocytes from the same liver, this method can provide a powerful tool to determine if hepatocytes derived from HSCs or iPSCs generated from HSCs can reproduce the key characteristics of hepatocytes from the same donor. If desired, iPSC-Heps derived from peripheral blood cells of the same donor can also be tested as it is generally possible to obtain a small volume of blood with written informed consent. This will allow researchers to identify the effects due to reprogramming to iPSCs and to understand the influence of different starting cell types for reprogramming. Another reason to publish this method is that although there are methods available to isolate HSCs, to our knowledge, there is no detailed protocol available for the isolation of high-quality HSCs and hepatocytes from the same donor.

## Conclusions

In conclusion, a method for the concurrent isolation of hepatocytes and HSCs has been successfully established. The method presented here is relatively fast as no additional enzymatic digestion steps have been added after the 2-step collagenase perfusion of the liver piece was complete. This brings the isolation time required down to a manageable time of approximately 6 h as livers donated from elective surgeries are usually received between early to late afternoon due to the time needed for the operations.

The isolation of HSCs and hepatocytes from the same donor will allow for the first time benchmarking of differentiated hepatocytes to the isolated hepatocytes from the same donor. In addition, when reprogramming to iPSC is undesirable, HSCs can also be maintained in culture for a prolonged period (Wang et al. 2010) for differentiation to hepatocytes. Furthermore, the effects of reprogramming to iPSCs can also be compared using HSCs due to their amenability to culture and subsequent direct differentiation to hepatocytes.

## Data Availability

The data used and/or analysed during the current study are available from the corresponding author on reasonable request and with permission from the authors in F. Hoffmann-La Roche Ltd.
